# In This Issue

**DOI:** 10.1002/cfg.384

**Published:** 2004-03


**Comparative and Functional Genomics**
*Comp Funct Genom* 2004; **5**: 115.
Published online in Wiley InterScience (www.interscience.wiley.com). **DOI:** 10.1002/cfg.384

### In this issue

### *Mycobacterium tuberculosis* DR region is deleted in several other mycobacteria

A region comprising the direct repeat (DR) region of the *M. tuberculosis* complex and 21 ORFs was found to be absent from the genomes of several other sequenced mycobacteria by **Karina Caimi** and **Angel Cataldi**.

### Three novel mouse pigmentation mutants

**Vicky Tsipouri** and **colleagues** recovered three novel mouse pigmentation mutants from an ENU mutagenesis study and determined the position of each of the causative mutations on the mouse genetic map.

### Phylogenetics in the bioinformatics culture of understanding

**Robin Allaby** and **Mathew Woodwark** argue that the way that bioinformaticians approach phylogenetic analyses often differs from that used by phylogeneticists, and show that some of the common phylogenetic questions tackled by bioinformaticians can be better addressed with a deeper understanding of evolutionary principles.

### Special Section of articles from the ESF Programme on Functional Genomics workshop on 'Data integration in functional genomics and proteomics: application to biological pathways'

**Pierre-Alain Binz**, **Paul van der Vet** and **Henning Hermjakob** open this special section with their review of the presentations and discussions from the meeting.

Tools for classification of chemical compounds and visualization of classified compounds have been designed to support complex queries in a pathway database by **Ulrike Wittig** and **colleagues**.

**Claudia Choi *et al.*** describe the signal transduction database, TRANSPATH, which has three modules: the manually extracted dataset; PathwayBuilder™, which provides network visualization; and Array Analyzer™, which is used for gene expression array interpretation.

**Paolo Romano** and **colleagues** discuss the interoperability of the Common Access to Biological Resources and Information (CABRI) services and biochemical pathway databases.

**Javier De Las Rivas** and **Alberto De Luis** describe existing protein interaction databases and the ways in which the datasets were obtained. They provide a definition of the different types of associations included in these datasets and briefly introduce a tool for viewing interaction networks, which will use the three levels of association that they have defined.

**Sergio Nasi** reviews current informatics resources and methodologies in the study of functional pathways in cell biology and discusses current approaches to model and simulate the dynamics of regulatory pathways in the cell.

**Heiko Schoof** and **co-workers** describe the objectives of, and progress made so far by, the PlaNet consortium, which is using a federated approach to provide a comprehensive plant genomic data platform.

The central dogma of molecular biology is used to guide the integration of genomic data but there is no such solid, well-known system for assembling proteomic data. **Frederique Lisacek** and **colleagues** describe their efforts to shape biological knowledge in this area.

In working to identify proteins in cancer-related 2D-PAGE maps, **Djamel Medjahed** and **colleagues** have developed VIRTUAL2D, a web-accessible repository of theoretical pI/Mw charts, and the Tissue Molecular Anatomy Project (TMAP), a set of tissue/histology-specific protein expression maps.

**Hans van Beek** highlights concerns about genomics becoming data-driven science and describes how the Centre for Medical Systems Biology's integration of datasets and data-mining approaches are being directed to generate hypotheses that are amenable to rapid testing in the laboratory.

### Conference calendar

Genomics-related conferences planned for July–September 2004.

Copyright © 2004 John Wiley & Sons, Ltd.

